# Characterisation of RSV infections in children without chronic diseases aged 0–36 months during the post-COVID-19 winter season 2022/2023

**DOI:** 10.3389/fped.2024.1342399

**Published:** 2024-02-06

**Authors:** Katharina Meier, Angela Riepl, Peter Voitl, Lena Lischka, Julian J. M. Voitl, Klara Langer, Ulrike Kuzio, Monika Redlberger-Fritz, Susanne C. Diesner-Treiber

**Affiliations:** ^1^ First Vienna Pediatric Medical Center, Vienna, Austria; ^2^ Sigmund Freud University Vienna, Vienna, Austria; ^3^ Center of Virology, Medical University of Vienna, Vienna, Austria

**Keywords:** RSV, genotypes, subtypes, children, acute respiratory infection, post-COVID-19, MTS score

## Abstract

**Background:**

Respiratory syncytial virus (RSV) is one of the leading causes of hospitalisation, morbidity, and mortality due to respiratory infection in the first years of life. This longitudinal prospective study outlines the 2022/23 season's viral patterns in Austria after the epidemiological changes determined by public health measures. We aimed to highlight differences within the RSV subtypes and genotypes in 0–36-month-old children without chronic diseases in the outpatient setting.

**Methods:**

From November 2022 to March 2023 children younger than 36 months admitted to Vienna's largest paediatric primary healthcare centre with an acute respiratory infection were enrolled in this study. Nasal swabs and multiplex PCR panels detected 20 viruses including RSV subtypes and genotypes. Clinical presentation, features, and treatment of the participants were documented and analysed using the Modified Tal Score (MTS). Patients were scheduled for a telemedical follow-up one week after the initial appointment. Analysis was done using descriptive statistics, including Cramér V and binominal logarithmic regression.

**Results:**

Among the 345 samples from 329 children, RSV was the most common virus (31.9%), followed by influenza (17.5%) and rhinovirus infections (20.58%). Of the RSV positive samples, only 13 cases were RSV subtype A (11.8%), whereas 97 were of subtype B (87.3%); ON1 and BA9 were the only detectable RSV genotypes (ON1: BA9 = 1:9.25). RSV was the main predictor of hospitalisation (OR: 7.5, 95% CI: (1.46–38.40), and age had a significant but smaller effect (OR: 0.89, 95% CI: (0.81–0.99). Almost all patients' clinical status improved within the first days.

**Conclusion:**

RSV cases showed a rapid onset in late November 2022, and subtype B was predominant throughout the season. RSV infection was associated with higher hospitalisation rates, even after excluding high-risk patients (preterm and severe chronic diseases population).

Further testing in the upcoming winter seasons will improve our knowledge of the dominant subtype and its association with disease severity, especially with the development of novel RSV vaccine candidates.

## 1 Introduction

Respiratory virus infections have been significantly impacted by the Covid-19 pandemic in recent years (1). They occur primarily in children 0–5 years of age and are one of the leading causes of hospitalisation, morbidity, and mortality in the first years of life worldwide (2). Influenza virus, rhinovirus, respiratory syncytial virus (RSV), human coronaviruses, parainfluenza virus, metapneumovirus, enterovirus, and adenovirus, are the most common viral pathogens associated with acute respiratory infections in childhood (3).

The Covid-19 pandemic altered the characteristic pattern of respiratory virus infections (1). The introduction of nonpharmaceutical interventions (NPIs), such as oral-nasal protection, hand hygiene, social distancing, travel restrictions, and school closures, as well as the emergence of new SARS-CoV-2 variants and the impact of Covid-19 vaccination campaigns, have determined significant variations in the epidemiology of these viruses (1).

In Austria, the implementation of protective measures against Covid-19 also led to a significant reduction in respiratory virus infections in 2020/21 (4). The following year, however, saw a substantial increase in respiratory virus infections as early as September and October 2021/22, particularly RSV (5).

RSV is one of the most frequent causes of mild upper respiratory tract infections to severe lower respiratory tract infections, such as pneumonia, bronchitis and bronchiolitis, in children 0–3 years of age (2, 6). Respiratory syncytial virus is a non-segmented orthopneumovirus with single-stranded RNA that belongs to the Pneumoviridae family. The attachment (F) and fusion (G) surface glycoproteins mediate the entry of the virus. RSV can be distinguished into two subtypes, RSV A and RSV B. Based on the genetic variability of the second hypervariable region (HVR2) of the G gene, RSV strains are further classified into genotypes. To date, while RSV A strains can be grouped in nine main genotypes, the literature describes 15 genotypes for RSV B strains (7–9). The distribution of RSV subtypes may vary over time and in different regions. The predominant RSV subtypes may change periodically, with different subtypes circulating in different years or seasons (8).

As the virus evolves, new variants continuously develop while others disappear. Whether and to what extent the two RSV types influence seasonality, transmission rate, rate of co-infection, and severity of virus-associated disease is controversially discussed in the literature (10–12).

Following our previous studies (4, 5), the aim of this monocentric longitudinal study was specifically to describe and characterize RSV infections in a low-risk pediatric population without severe chronic diseases. We aimed to highlight differences between RSV subtypes and genotypes in children aged 0–36 months in the outpatient setting during a winter season after the Covid-19 pandemic. We investigated RSV as predictor of hospitalization and described possible differences in co-infections, treatment plans, and seasonal variations. In addition, symptom severity of RSV positive and negative cases was described using the modified Tal score (MTS).

## 2 Materials and methods

### 2.1 Study design

The study was conducted at the primary health care centre “First Vienna Pediatric Medical Center.” The recruitment phase took place from November 2022 to March 2023, the typical RSV infection season in Austria before Covid-19 (13). Children without chronic infections, younger than the complete 36 months of age (≤3rd birthday), who presented with at least one of doctor's diagnosed acute respiratory infection symptoms (cough, rhinitis, nasal congestion, sore throat/pharyngitis, fever, otalgia) were eligible for this study. Fever was predefined as body temperature of at least 38 °C measured with an ear thermometer. In order to prevent a selection bias, swabs were taken on different days of the week during the normal practice opening hours (in total 66 days over the recruitment period) because due to limited resources not all of the eligible patients could be included. It was the aim to include as many patients as possible and at least 100 RSV positive children.

The following exclusion criteria were based on the RSV high-risk profile according to the RSV prevention guideline (14): preterm birth (<37 weeks of gestation), severe congenital pulmonary diseases (e.g., cystic fibrosis, primary ciliary dyskinesia, interstitial lung diseases), neuromuscular diseases with impaired lung clearance, severe hemodynamic cardiac diseases, or severe immunodeficiency (e.g., immunoglobulin deficiency); and in addition, older children (age >36 + 0 months).

The study design remains as described in the publications of the preceding seasons of 2020/21 and 2021/22 (4, 5), except for the following changes: the multiplex PCR was done at the Medical University of Vienna, Center of Virology within the RSV surveillance network project (13). Additionally, RSV subtypes and genotypes were characterised (15).

Telemedical follow-up was conducted one week after the first medical appointment to discuss the results and changes in the clinical status.

The patient's general clinical data (height, weight, chronic diseases), doctor's diagnosed acute respiratory symptoms (rhinitis, nasal congestion, cough, fever, pharyngitis), doctor's diagnosis (otitis media, bronchitis, bronchiolitis, pneumonia, upper airway infection (rhinitis, nasal congestion, throat infection), others (fever without further diagnosis, not further specified diagnosis), and general state of health [respiratory rate, oxygenation (SpO2), wheezing/crackles, accessory respiratory muscle utilisation] were documented in a case report form. Furthermore, prescribed symptomatic treatment (hypertonic saline spray, decongestant nasal spray, analgesics, antibiotics, corticosteroid treatment (inhaled or systemic), inhalation therapy of short-acting betamimetics (SABA) or 0.9% sodium chloride) were documented as well.

Parents signed the declaration of consent after being informed about the research aims and the study procedure.

The following data were gathered during the telemedical follow-up by the study team: respiratory symptoms and treatment, parent's subjective evaluation of the child's health (improved, deteriorated, or unchanged), and up-date on hospitalisation due to the respiratory infection within the last week.

Furthermore, information regarding the family's socioeconomic status (living situation, marital status, parent´s education, number of siblings) was determined by interviewing the corresponding parent either at the day of study inclusion or during the telemedical follow up control.

This study was approved by the Ethics Committee of the Medical University of Vienna (EK-No. 1864/2020). Due to the national regulations it was not possible to collect children's data without consent (e.g., total number of eligible children), as this violates the General Data Protection regulation.

### 2.2 Study procedure

During the doctor's appointment, a trained study team collected anterior nasal swabs. The samples were stored in 0.9% sodium chloride solution until further analysis via multiplex PCR. The following viral pathogens were tested: RSV including RSV-subtype and genotype, rhinovirus, adenovirus, metapneumovirus, enterovirus, SARS-CoV-2, human coronaviruses (229E, HKU1, OC43, NL63), influenza A (H1 2009, H3), influenza B (Yamagata, Victoria), influenza C, and parainfluenza virus 1–3.

The PCR evaluation and the RSV subgroup analysis were performed at the Center for Virology, Medical University of Vienna, and incorporated into the statistical report of the RSV Surveillance Network (13).

In case of recurrent acute respiratory infection in one patient, nasal swabs were repeated as long as there was a symptom-free interval of at least one week between visits.

To assess the severity of respiratory disease the Modified Tal Score (MTS) was used. This clinical ordinal scoring system has been used in multiple publications and is validated to categorise bronchiolitis especially among RSV infections and the severity of respiratory disease (16–18). Points were assigned depending on accessory muscle use (none (0 points)/mild/moderate/severe (3 points)), respiratory rate (<6 months of age: 0 points: <40, 1: 41–55, 2: 56–70; 3 points:>70/min; >6 months of age: 0: <30, 1: 31–45, 2: 46–60, 3 points > 60/min), degree of wheezing or crackles (none: 0 points/end expiratory with stethoscope/inspiration and expiration with stethoscope/3 points: audible without stethoscope), and SpO2 values (0 points: SpO2 > 95%, 1: 92%–94%, 2: 90%–91%, 3: <89%). Therefore, 0 points indicated no bronchiolitis, 1–5 points suggested a mild infection, 6–10 points a moderate one, and 11–12 points severe disease.

### 2.3 Statistics

The principal aim of the analyses was to describe and characterize RSV infections over the winter season and the severity and risk for hospitalization in comparison to non-RSV and negative tested patients. In addition, we analysed the clinical course, the symptoms, prescribed therapy and the doctor's diagnosis.

Raw data was sorted in Google Sheets and Microsoft Excel and analzyed using IBM SPSS-Statistics version 29 and GraphPad Prism version 10. A two-sided *p* value of *p* < 0.05 indicated statistical significance. The Bonferroni test corrected the risk of higher frequency errors due to multiple testing, resulting in adjusted *p*-values (*p*-adj.).

Most data were categorised as nominal variables. Metric variables (age, weight, height, BMI, number of siblings) were used to describe the study population further.

Due to the low number of specific pathogens, the following groups were built: single infections with RSV, adenovirus, rhinovirus, enterovirus, metapneumovirus, human coronaviruses, influenza and parainfluenza. Co-infections constitute the category “co-infection”, and results without a detected viral pathogen the group “negative”.

Qualitative variables such as patient characteristics, manifestation of symptoms, recommended therapy, doctor's diagnosis and pathogen groups, as well as the comparison of these variables, were presented as descriptive statistics through absolute and relative frequency (percentages). As for metric variables, mean, median, interquartile range, and first and third quartiles were calculated.

The statistical analysis consists mainly of descriptive analysis. The sociodemographic data were reported for the entire study population using descriptive statistics.

Nominally and ordinally scaled variables such as pathogen groups/RSV subtypes, symptoms, treatments, diagnosis, and MTS were tested using the Chi-Square test or Fisher's Exact test. Associations of pathogen groups and symptoms, as well as pathogen group and treatment were analysed in contingency tables using the effect size Cramér V, which was interpreted using the Rea and Parker's classification (*r* = 0.10–0.20 weak, *r* = 0.20–0.40 moderate, and *r* = 0.40–0.60 relatively strong association) (19).

For detailed RSV characterization, we generated four groups: RSV A, RSV B (single infections only), non-RSV (single infections), and negative (negative PCR results). Co-infections were not included in this analysis. Sociodemographic data were reported for these groups. Associations of these groups and symptoms, treatment, severity (no bronchiolitis, mild-moderate-severe according to MTS) and state of health (improved, deteriorated, or unchanged) at the telemedical follow-up control were analysed in contingency tables using Cramér V. All *p* values were corrected using the Bonferroni method for multiple testing.

A binominal logistic regression was performed to ascertain the effects of RSV detection (yes/no, independent on the co-infection status), age (in months), gender (male/female), and MTS score (0–12) (all independent variables) on the likelihood of hospitalization [(yes/no); dependent variable]. The omnibus test of the model coefficients indicated the significance of the model and the Nagelkerke R2 was used to interpret the amount of explained variance. Wald test was used to test for significance and odds ratio (OR), 95% confidence interval (95% CI) and *p*-values were calculated.

## 3 Results

### 3.1 Study population and pathogen characterization

**Figure 1:**
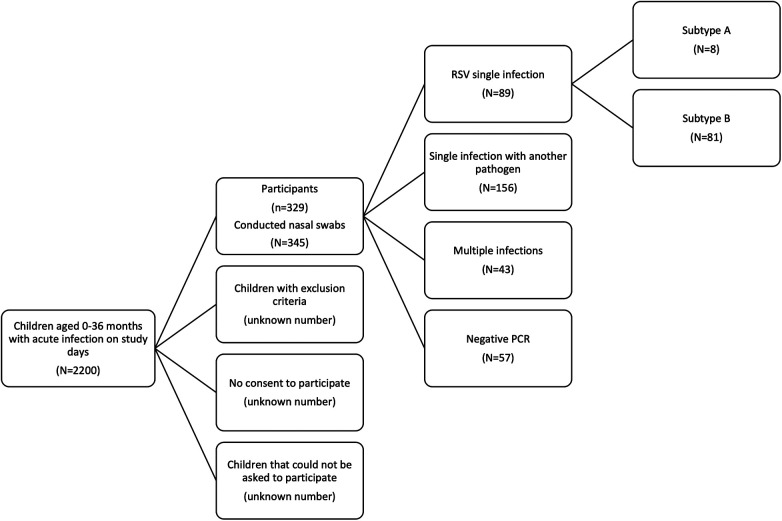
Flow chart of inclusion procedure. The inclusion process of children younger than 36 months with an acute infection is depicted. Out of the 345 conducted nasal swabs, we detected RSV single infections (*N* = 89), single infections other than RSV (*N* = 156), multiple infections (*N* = 43) and had in 57 swabs negative PCR results.

Swabs were performed on 66 days throughout the study period. On these days, a total of 2,200 acutely ill children aged 0–36 months presented to the practice (Figure 1). After applying the inclusion and exclusion criteria, 329 children with acute respiratory infections participated in the study from November 2022 to March 2023. The median age was 14 months (Q1–Q3: 6.5–23.5 months); 47.4% of patients were male and 52.6% female. In total, 345 nose swabs were collected. During the study period, 314 children had one swab, 14 children had two swabs, and one child had three nose swabs. All sociodemographic and general clinical characteristics are summarised in Table 1.

**Table 1:** Characterization of study population.

		Total study population	RSV A	RSV B	Non-RSV	Negative
Age	Months	14.0 (6.5–23.5)	18.0 (6.3–27.3)	11.0 (6.0–21.0)	15.5 (7.0–24.0)	11.5 (5.8–22.5)
BMI	Kg/m^2^	16.1 (14.8–17.3)	16.4 (15.3–17.4)	16.0 (14.9–17.2)	16.1 (14.7–17.2)	16.4 (14.8–17.3)
Sex	Male	156 (47.4)	4 (50.0)	33 (40.7)	68 (45.9)	33 (61.1)
	Female	173 (52.6)	4 (50.0)	48 (59.3)	80 (54.1)	21 (38.9)
Siblings	Number	1 (0–1)	1 (0–2)	1 (0–1)	1 (0–1)	0 (0–1)
	Missing data	6 (1.8)	0 (0.0)	1 (1.2)	4 (2.7)	1 (1.9)
Highest education mother	Compulsory education	17 (5.2)	0 (0.0)	6 (7.4)	8 (5.4)	1 (1.9)
	Apprenticeship	63 (19.1)	1 (12.5)	15 (18.5)	27 (18.2)	8 (14.8)
	High school diploma	88 (26.7)	1 (12.5)	24 (30.9)	34 (23.0)	21 (38.9)
	Academic degree	139 (42.2)	5 (62.5)	32 (39.5)	67 (45.3)	20 (37.0)
	Missing data	22 (6.7)	1 (12.5)	4 (4.9)	12 (8.1)	4 (7.4)
Highest education father	Compulsory education	11 (3.3)	0 (0.0)	7 (8.6)	2 (1.4)	0 (0.0)
	Apprenticeship	97 (29.5)	1 (12.5)	24 (29.6)	40 (27.0)	20 (37.0)
	High school diploma	69 (21.0)	1 (12.5)	16 (19.8)	32 (21.6)	14 (25.9)
	Academic degree	125 (38.0)	5 (62.5)	29 (35.8)	59 (39.9)	16 (29.6)
	Missing data	27 (8.2)	1 (12.5)	5 (6.2)	15 (10.1)	4 (7.4)
Parent's marital status	In relation	311 (94.5)	7 (87.5)	76 (93.8)	143 (96.6)	51 (94.4)
	Separated	17 (5.2)	1 (12.5)	4 (4.9)	5 (3.4)	3 (5.6)
	Missing data	1 (<1)	0 (0.0)	1 (1.2)	0 (0.0)	0 (0.0)
Housing	Community housing	37 (11.2)	2 (25.0)	10 (12.3)	11 (7.4)	8 (14.8)
	Apartment	232 (70.5)	4 (50.0)	60 (74.1)	106 (71.6)	34 (63.0)
	House	58 (17.6)	2 (25.0)	10 (12.3)	31 (20.9)	12 (22.2)
	Missing data	2 (<1)	0 (0.0)	1 (1.2)	0 (0.0)	0 (0.0)
Chronic diseases	Yes	8 (2.4)	0 (0.0)	2 (2.5)	3 (2.0)	3 (5.6)
	No	320 (97.3)	8 (100.0)	78 (96.3)	145 (98.0)	51 (94.4)
	Missing data	1 (<1)	0 (0.0)	1 (1.2)	0 (0.0)	0 (0.0)
Total cases		329	8	81	148	54

Metric data are presented as median and (Q1–Q3), qualitiative values as absolute numbers and relative frequencies in percentage per column (%). *N* = 329.

Single infections for RSV A, RSV B and Non-RSV infections are shown. Co-infections are included in the category “total study population”. There are no significant differences between the groups.

The most common clinical symptom was cough (84.6%), followed by rhinitis (73.0%) and fever (53.3%) (Table 2). Patients were treated with analgesics (70.4%), decongestant and hypertonic saline nose sprays/drops (62.0%), short-acting betamimetics (13.9%), and antibiotics (13.3%). Of the 345 included visits, 291 cases received one clinical diagnosis (84.3%), 48 received two (13.9%) and 6 got three (1.7%). Most participants (279 of 345 cases, 80.9%) were clinically diagnosed by their attending physician with an upper airway infection, independent of the PCR result, followed by bronchitis (47/345, 13.6%) and otitis media (42/345, 12.2%) (details see Table 2).

**Table 2:** Clinical characterization, diagnosis and medication.

	Total number of cases	RSV A	RSV B	Non-RSV	Negative
Clinical characteristics					
Cough	292 (84.6)	8 (100.0)	78 (96.3)	123 (78.8)	45 (78.9)
Rhinitis	252 (73.0)	7 (87.5)	61 (75.3)	108 (69.2)	42 (73.7)
Nasal congestion	49 (14.2)	1 (12.5)	14 (17.3)	18 (11.5)	10 (17.5)
Fever	184 (53.3)	6 (75.0)	37 (45.7)	91 (58.3)	28 (49.1)
Pharyngitis	99 (28.7)	2 (25.0)	28 (34.6)	46 (29.5)	14 (24.6)
Prescribed medication					
Decongestant and hypertonic saline nose spray	214 (62.0)	6 (75.0)	58 (71.6)	85 (54.5)	32 (56.1)
Analgesics	243 (70.4)	6 (75.0)	48 (59.3)	121 (77.6)	37 (64.9)
Inhalation SABA^b^	48 (13.9)	1 (12.5)	23 (28.4)	12 (7.7)	4 (7.0)
Antibiotics	46 (13.3)	2 (25.0)	6 (7.4)	27 (17.3)	5 (8.8)
Systemic corticosteroids	25 (7.2)	0 (0.0)	11 (13.6)	9 (5.8)	3 (5.3)
Inhaled corticosteroids	10 (2.9)	1 (12.5)	1 (1.2)	4 (2.6)	1 (1.8)
0.9% sodium chloride inhalation	7 (2.0)	1 (12.5)	3 (3.7)	0 (0.0)	2 (3.5)
Doctor's diagnosis^a^					
Otitis media	42 (12.2)	2 (25.0)	13 (16.0)	14 (9.0)	5 (8.8)
Bronchitis	47 (13.6)	2 (25.0)	21 (25.9)	12 (7.7)	4 (7.0)
Bronchiolitis	11 (3.2)	0 (0.0)	6 (7.4)	2 (1.3)	0 (0.0)
Pneumonia	6 (1.7)	0 (0.0)	2 (2.5)	3 (1.9)	1 (1.8)
Upper respiratory tract infection	279 (80.9)	5 (62.5)	57 (70.4)	137 (87.8)	47 (82.5)
Other diagnosis	20 (5.8)	0 (0.0)	5 (6.2)	8 (5.1)	5 (8.8)
Total cases	345	8	81	156	57

Data are presented as absolute number and relative frequency (%). Children had multiple symptoms and diagnosis and were prescribed multiple medications; therefore, multiple responses are possible. *N* = 345.

Single infections are shown for the categories RSV A, RSV B and Non-RSV. Co-infections are included in the category “total number of cases”.

^a^ Clinical diagnosis by the attending physician.

^b^ SABA: short acting beta mimetics.

**Figure 2:**
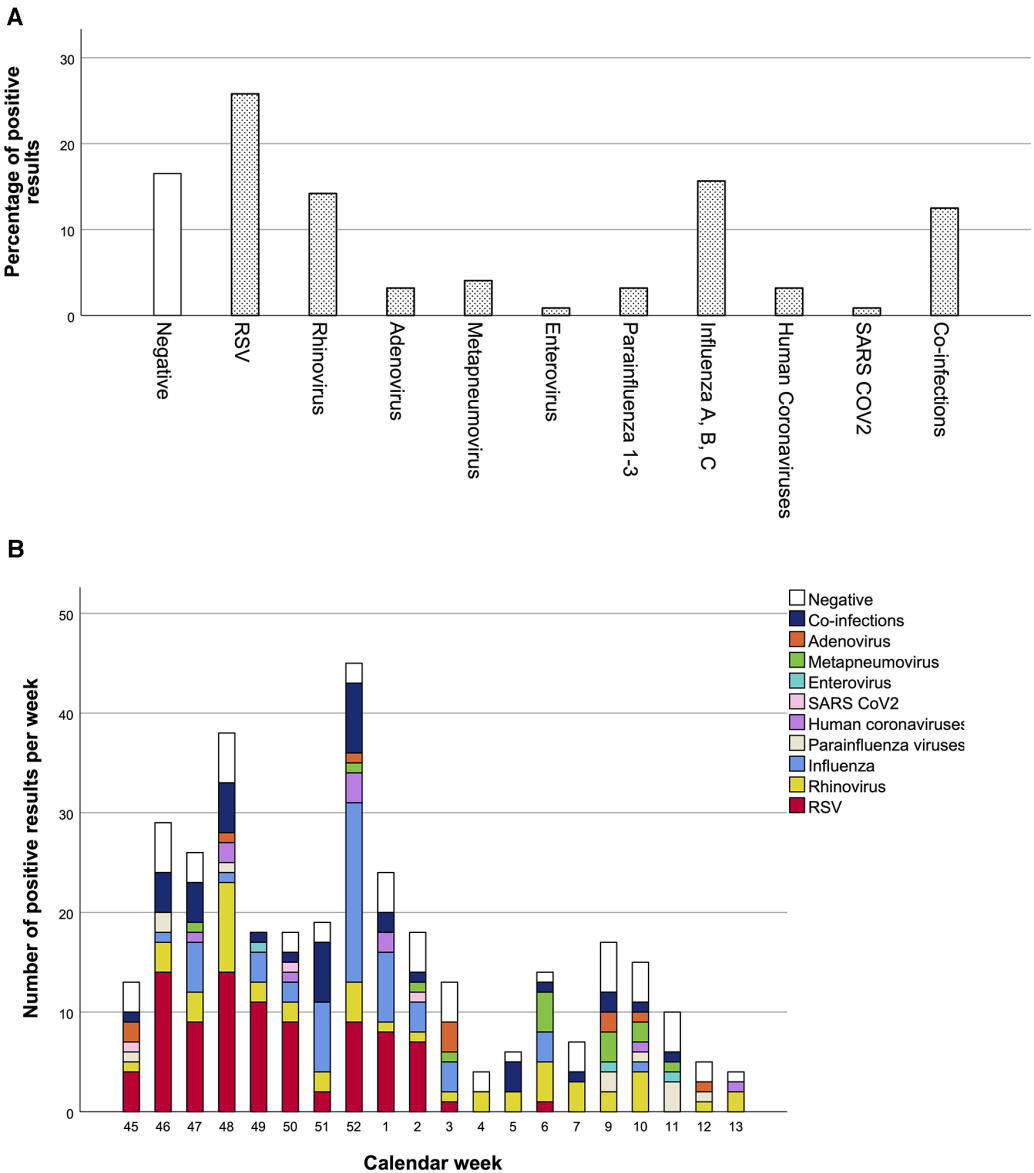
Percentage of positive pathogen result and distribution of pathogens over the winter season. Percentages of positive results over the whole winter season are shown in A, and detection rates per calendar week in B. Total number of nasal swabs: 345.

Co-infections were found in 43 samples (12.5%) (41 of these with two pathogens and two with three pathogens), while no viral pathogen was detected in 57 cases (16.5%). The most common virus was RSV (110/345, 31.9%), with 89 single infections (25.8%), and 21 cases of co-infections including RSV (6.1%) (Figure 2A). The highest incidence of positive results and RSV cases was registered in weeks 46–52 (Figure 2B), followed by a decline with sporadic RSV diagnoses. In addition to RSV, influenza (17.5%) and rhinovirus infections (20.58%) were very common. The influenza vaccination rate was 19.5%.

Comparing the frequency of symptoms and all tested pathogen groups (including multiple viral infections and negative PCR results as separate category) revealed a significant and moderate association (Cramér's V: 0.346, *p* < 0.001, *p*-adj. <0.01) (Supplementary Table 1). The highest frequency of cough and rhinitis was found among RSV cases (96.6% and 76.4%), human coronaviruses (100% and 90.9%), parainfluenza (90.9% and 81.8%), and rhinovirus cases (85.7% and 81.6%). Fever was most common among individuals with adenovirus (90.9%), enterovirus (100%), and influenza infections (85.2%), whereas pharyngitis was mostly found in children with enterovirus (66.7%) and parainfluenza 1–3 infections (54.5%).

In contrast, no significant association emerged between prescribed treatment and pathogen group (Cramér V: 0.298, *p* = 0.396, *p*-adj. >0.999).

### 3.2 RSV characterisation

Out of the 110 positive RSV results (including co-infections), only 13 cases were subtype A (13/110, 11.8%), 96 were subtype B (96/110, 87.3%), and one RSV subtype could not be determined (<1%). Eight of the 13 RSV subtype A cases were of the ON1 genotype (8/110, 7.3%), 74 of the RSV subtype B cases were of the BA9 genotype (74/110, 67.3%), and in 28 cases, the genotype could not be identified (28/110, 25.5%). No other genotypes were detected.

**Figure 3:**
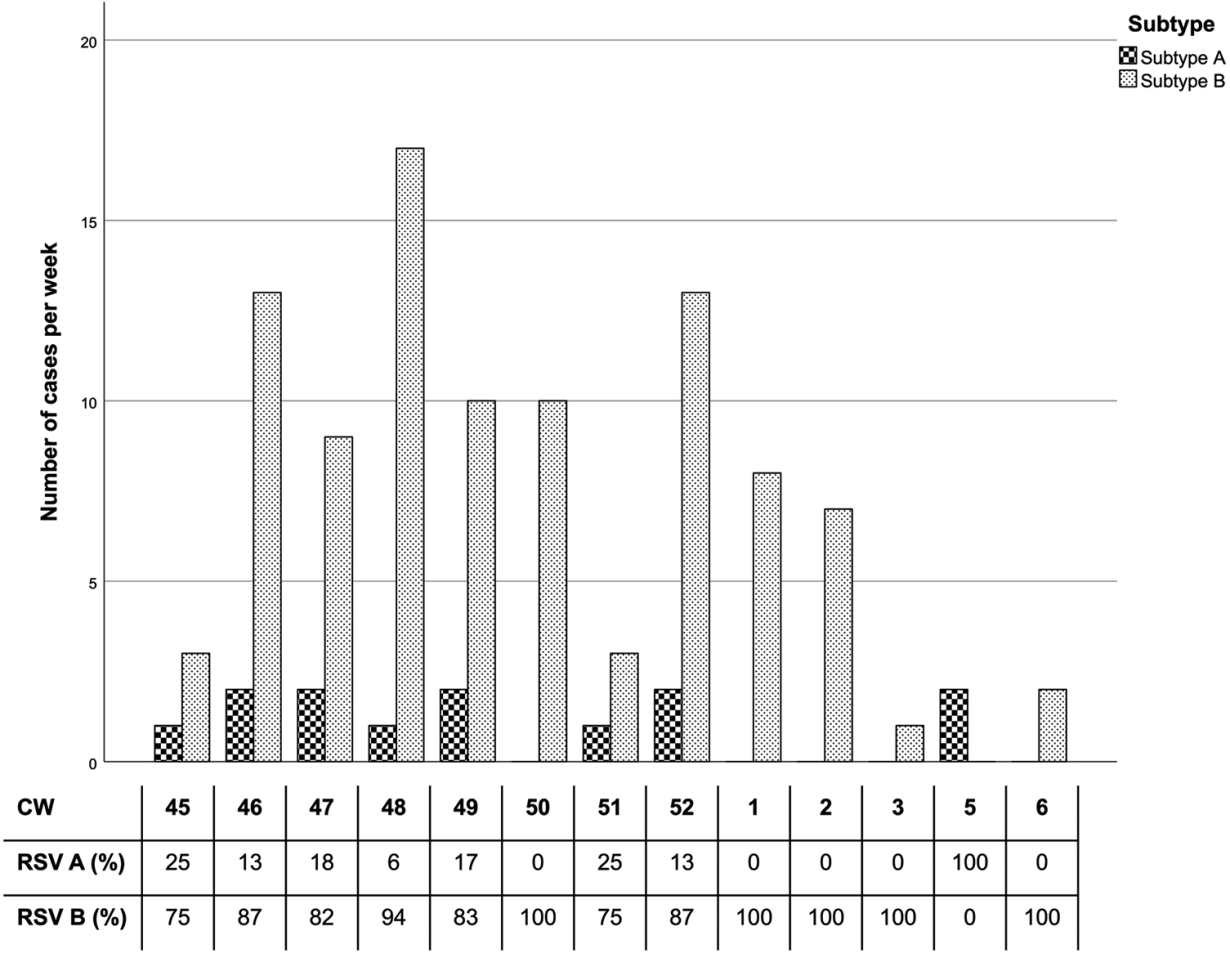
RSV subtype per calendar week. The percentages reflect subtype distribution of positive RSV cases each week. Only CWs (calendar weeks) with RSV cases are included. CW 4 and after CW 6 data did not register positive RSV cases.

Comparing occurrence of RSV cases and distribution of subtypes (Figure 3), the peak in RSV cases, specifically among subtype B patients, was recorded during the calendar weeks 46 to 2 (especially week 48), whereas subtype A cases were steadily low with 0–3 positive results per week throughout the season.

Four pathogen groups were established to examine correlations between participants’ symptoms, prescribed treatment, doctor's diagnosis, MTS and the children's state of health one week later at the follow-up appointment: RSV A, RSV B, non-RSV (infections without RSV), and negative PCR results. Only cases of single infections (absence of co-infection) were considered among those four groups. No significant differences of sociodemographic data were observed between these groups (Table 1).

Symptoms of patients with RSV subtype A and B were very similar, except for fever which was more common in the RSV A group (75.0% vs. 45.7% in RSV B). The participants' recorded symptoms revealed a moderate but not significant correlation with the corresponding groups. (Cramér V: 0.322, *p* = 0.068, *p*-adj. 0.612). Conversely, a moderate and significant correlation can be observed between participants’ treatment plans and the four groups (Cramér V: 0.380, *p* < 0.001, *p*-adj. <0.01) with a high proportion of RSV B patients being prescribed SABA inhalation (Supplementary Table 1). RSV A patients mainly received nose drops or analgesics. No significant differences were observed with regard to doctor's diagnosis between the four groups (Cramér V: 0.260, *p* = 0.235, *p*-adj. >0.999). Twenty-five percent of RSV single infection cases received the diagnosis of bronchitis (RSV A: 2 out of 8, RSV B: 21 out of 81); and 54.5% of all diagnosed bronchiolitis cases were infected with an RSV B single infection (6 out of 11 cases) (Table 2).

Comparing the MTS as a tool to ascertain the severity of bronchiolitis with the cases of RSV A, RSV B, non-RSV infections, and negative tests reveals a moderate correlation (Cramér V: 0.316, *p* < 0.001, *p*-adj. *p* < 0.01).

**Figure 4:**
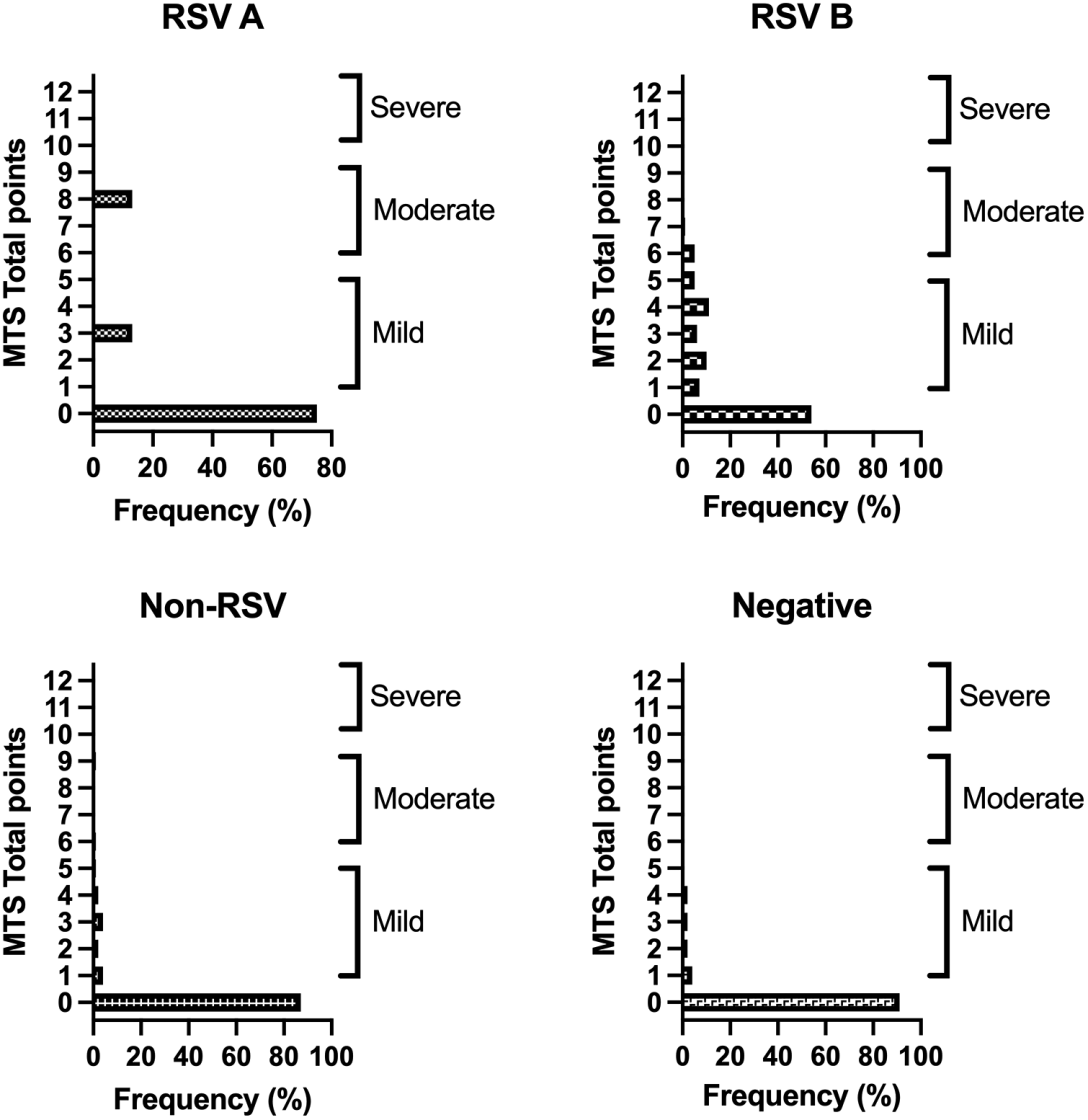
Results of the MTS of RSV subtypes and non-RSV cases. Depicted percentages are calculated within the four groups (subtype A, B, non-RSV and negative group excluding cases of multiple infections). MTS 0 points: no bronchiolitis, MTS 1–5: mild bronchiolitis, MTS 6–10: moderate bronchiolitis, MTS 11–12: severe bronchiolitis.

Out of the 89 RSV single infections, most participants infected with either subtype A or B showed no signs of bronchiolitis (Figure 4) or mild bronchiolitis, according to the MTS. One RSV A and 5 RSV B patients suffered from moderate bronchiolitis. There were no cases of severe bronchiolitis among our study subjects. Almost all children with a negative PCR test result or an infection without RSV, showed no sign of bronchiolitis (Table 2).

A logistic regression was performed to ascertain the effects of age, gender, MTS, and RSV on the likelihood that children are hospitalised. The logistic regression model was statistically significant [*X*2(4) = 27.68, *p* < 0.001]. The model explained 28% (Nagelkerke R2) of the variance in hospitalisation rate and correctly classified 96.1% of cases. RSV positive patients were 7.5 times (95% CI: 1.46–38.40) more likely to be hospitalised than patients without RSV. The odds of hospitalization was significantly lower with increasing age. A higher MTS score was trendwise (*p* = 0.08) associated with a higher hospitalization risk which, however was not significant (Table 3).

**Table 3:** Predictors of hospitalization (*N* = 336).

	OR	95% CI	*p*-value
RSV	7.45	(1.46–38.40)	0.016
Age (in months)	0.89	(0.81–0.98)	0.027
Gender	0.98	(0.30–3.24)	0.976
MTS score	1.27	(0.97–1.66)	0.080

Binary logarithmic regression: dependent variable hospitalization, independent variables (gender, RSV, age (in months), MTS score. Total number: *N* = 345 including multiple infections, 9 missing cases; for calculation *N* = 336.

In nine cases the final data of hospitalization are missing because the telemedical follow-up was not possible. In general, thirteen hospitalisations occurred because of a respiratory infection during the observed interval (13/336, 3.9%). Out of these 13, in nine cases hospitalisation was necessary due to RSV single infections: one case of RSV subtype A and eight cases of RSV B. No participant infected with another single pathogen was admitted to the hospital. In two cases the hospitalised child was infected by multiple pathogens, in both cases including RSV (in combination with adenovirus and coronavirus OC43). In the last two cases of hospitalisation the pathogen was not detectable (PCR negative).

The one-week follow-up confirmed that the severity of symptoms had improved in almost all 110 RSV positive cases (including co-infections), non-RSV infections, and negative cases (100 out of the 110 RSV cases) with no significant difference within the RSV subtypes, non-RSV, and negative cases. (Cramérs V = 0.119, *p* = 0.214 *p*-adj. >0.999).

## 4 Discussion

In recent years, our goal has been to describe the course of illness, the severity of symptoms, and treatment of infants and young children up to the age of 3 with acute respiratory infection in Vienna's largest paediatric primary care centre, the first point of contact for parents with acutely ill children. This longitudinal study aimed to examine the post-Covid winter season 2022/2023 and focused on RSV distribution, subtypes, and association with severity of illness and hospitalisation rates.

Our findings proved RSV to be the most common ailment among our patients, followed by rhinovirus and influenza. RSV distribution peaked in the last calendar weeks of 2022, with the RSV B being the predominant subtype. Genotyping revealed only two predominant RSV genotypes: ON1 for RSV A and BA9 for RSV B. Most patients only had mild bronchiolitis and occasionally moderate-severe bronchiolitis as diagnosed by MTS. The risk for hospitalization was significantly higher when RSV was present. A higher MTS score had a trendwise effect on hospitalization but was not significant. In conclusion, an RSV infection represents a risk factor for hospital admission in RSV low-risk children without chronic infections. Most patients showed a rapid improvement in their health within a few days.

Covid-19 has had a powerful impact on the prevalence of RSV internationally. While in 2020/2021, the coronavirus regulations disrupted the spread of RSV, the following winter season saw a severe and premature RSV surge in Austria (4, 5). This phenomenon appeared to slow down in the 2022/2023 season despite the earlier onset compared to pre pandemic years (13). Hence, the initial curb of infections in 0–36-month-olds could have potentially resulted in this wave of RSV cases in late November/early December 2022 and increased hospitalisation rates, as also recorded by the US RSV Network (20, 21).

The literature offers limited data on the severity and course of RSV infections in primary health care settings, as many studies have focused on the outpatient setting or hospitalised patients (2, 10, 11, 21, 22). In the first years of life, infants face a significant burden due to RSV in both inpatient and outpatient settings (23–25). The healthcare burden of RSV in healthy term-born infants in Europe is substantial. The incidence of RSV-associated hospitalizations is between 1% and 8% in the first year of life. This means that one in 56 healthy term-born infants is hospitalized annually due to RSV (26). According to the study by Wilderbeest et al., the largest prospective birth cohort to determine RSV burden was a South African single-center study. In this study, 54 RSV-associated hospitalizations were documented in 1,143 children in the first two years of life (26). RSV does not only affect children's and families' life during the acute infection, large population-based studies show a long-term association of RSV infections during infancy and childhood asthma (27). It is therefore essential to better characterize the occurrence of RSV in children in the outpatient setting, too, so that the patients can receive better care in the long term.

We resorted to the Modified Tal Score (MTS) as a standardised and validated system to assess the severity of RSV bronchiolitis. The MTS is a simple tool to evaluate the course of illness and oxygen requirements of RSV paediatric patients, which all physicians can use (16, 28, 29). Furthermore, it was shown that the MTS is not only applicable to RSV bronchiolitis, but also works validly for other respiratory disorders (16–18). Regrettably, almost no data exists on its use in primary healthcare.

Our patients suffered from mild RSV bronchiolitis, corresponding to a low MTS score (1–5 points). Only a small percentage had a moderate infection (MTS 6–10 points). Ten percent (11/110) of all RSV patients required hospitalisation, a greater number than among non-RSV patients (2/226, 0.8%, with nine missing cases). Our data do not suggest a subdued 2022/2023 RSV season.

As our patient population comprised otherwise healthy children who did not match the high-risk RSV profile, we anticipated a milder disease course. In addition, we can assume that children with acute lower respiratory tract infections would be directly admitted to hospitals. Nevertheless, in otherwise healthy toddlers and infants, an RSV infection leads to a 7.5-fold increased likelihood of hospitalisation.

Regarding the severity of respiratory infection, symptoms, treatment, and disease progression, the influence of RSV subtypes remains unclear, and data generated in primary healthcare centres are insufficient. We observed more subtype B cases (96/110, 87.3%) than subtype A (13/110, 11.8%) throughout the season, in line with numerous international studies indicating alternate predominance patterns. Rarely can a change in subtype dominance be detected at the end of a season (15, 22, 30, 31).

The low number of RSV A cases did not allow the analysis for statistically significant differences between the subtypes regarding the severity of the respiratory infection. This study found no correlation between the RSV subtype and participants' symptoms and symptomatic treatment or hospitalisation rates. Other studies provide inconsistent findings. Gilca et al. found that subtype A was associated with higher disease severity, higher frequency of fever, increased heart rate, and required oxygen support (>30%) (32). McConnochie et al. linked subtype A to more severe cases of RSV, especially among high-risk infants (33), while Midulla et al. associated it with a higher respiratory frequency and more frequent chest retractions (34). On the contrary, other research efforts found no association within the subtypes (35–38). Nevertheless, comparisons are difficult to make due to diverse definitions of disease severity. Some publications suggest that the genotypes rather than the subtypes determine the differing courses of disease and the varying results (34, 35). Our tests detected two genotypes: ON1 (subtype A) and BA9 (subtype B). Other studies conducted in Italy, Austria, and the USA concluded that, during the 2021/22 and 2022/23 outbreaks, ON1 and BA were the predominant strains circulating exclusively within the associated subtype. It is impossible to build a hypothesis regarding the influence of genotypes from our data due to the low number of RSV A cases. De facto, new variants constantly evolve, whereas others disappear (15, 22, 39, 40).

The question whether there is a difference between RSV subtypes regarding variables like respiratory symptoms and severity of infection needs to be addressed in further seasons in the outpatient setting to achieve more robust results. New RSV vaccines for a broader range of patients will open new scenarios on RSV infections among very young patients, the RSV subtypes, disease severity, and hospitalisation rates.

In autumn 2023, the RSV vaccine Abrysvo™ was approved in Austria for pregnant women between the 24th and 36th week of pregnancy (41). Immunization of the mother leads to passive transfer of maternal antibodies via the placenta to the fetus to prevent RSV disease in early infancy (42–44). This happens before an active vaccination can generate an effective immune response in infants leading to a protection directly after birth (45).

Nirsevimab, a monoclonal antibody against RSV with an extended half-life, was recently approved in Europe for the prevention of RSV lower respiratory tract disease in neonates, infants and young children during their first RSV season and will be on the market in 2024 in many countries (41, 45, 46). To prevent RSV lower respiratory tract disease in infants and children up to 24 months of age, a single injection is recommended to provide protection throughout the RSV season (47). Thereby, infection rates and hospitalizations associated with RSV in both healthy term and preterm infants are significantly reduced (47, 48).

The recently approved vaccines have the potential to reduce the burden of disease and mortality in children and the elderly. An additional advantage is that they only need to be administered once, which could increase acceptance in the population. Integrating these vaccines into routine immunization guidelines could help reduce RSV-related hospitalizations and deaths and contribute to improved disease outcomes (49). However, it will be crucial to develop global guidelines and national strategies to ensure the successful implementation of RSV vaccination. In addition, health systems must ensure that RSV vaccines are available in terms of affordability and accessibility, particularly in low- and middle-income countries. The provision of further trial data and comprehensive post-market surveillance of RSV vaccines will help to build confidence in long-term efficacy and safety (50).

### 4.1 Limitations

Although the “First Vienna Pediatric Medical Center” serves the highest annual number of patients of every socioeconomic status in Vienna, the monocentric design of this study is a limiting factor and could have potentially influenced its outcome. A national record of all RSV-positive cases from primary healthcare settings and hospitals would lead to more comprehensive data collection. The opportunity to participate in this study was generally well received; nevertheless, we lack the number of potential participants who decided not to be involved. Due to data protection regulations we are not able to specify the numbers of excluded patients.

As for the socioeconomic status of our population, participants with parents with university degrees are overrepresented compared to Vienna's general public. This factor could have potentially led to differing social circumstances in the study population regarding income, housing situation or number of siblings.

Anterior nasal swabs were preferred to the more invasive nasopharyngeal swabs as they reportedly provide valid results (51); they can be however, potentially inaccurate.

The Modified Tal Score, used to assess the severity of bronchiolitis, is validated and has been used in multiple publications especially for RSV bronchiolitis. However, it was shown that the MTS is not only applicable to RSV bronchiolitis, but also works validly for other respiratory disorders, although the data is limited.

## 5 Conclusion

Evidence shows that the RSV season is gradually returning to its pre-pandemic pattern and remains the most common pathogen in our acutely ill patients younger than 36 months. In our pediatric primary healthcare center, we mainly observed mild cases caused by RSV B. Nevertheless, RSV is still leading to hospitalisations significantly more often than any other pathogen among patients this age, even without increased risk of severe RSV progression. With new vaccines available for a broader population, data collection will expand our understanding of the disease course in the primary healthcare setting.

## Data Availability

The raw data supporting the conclusions of this article will be made available by the authors, without undue reservation.
