# Supplementary material for: Characterisation of RSV infections in children without chronic diseases aged 0–36 months during the post-COVID-19 winter season 2022/2023
Source: Front Pediatr. 2024 Feb 6;12:1342399. doi: 10.3389/fped.2024.1342399 (PMC10876782; doi:10.3389/fped.2024.1342399)
Supplement: Supplementary file 1 [file Table1.docx]

**Supplementary material**

**Supplementary Table 1.** Frequency of symptoms and treatment per pathogen category

|  | **Symptoms** | | | | | **Treatment** | | | | | | |
| --- | --- | --- | --- | --- | --- | --- | --- | --- | --- | --- | --- | --- |
|  | Cough | Rhinitis | Nasal congestion | Fever | Pharyngitis | Nasal spray | Analgesics | Inhaled SABA | Antibiotics | Systemic cortison | Inhaled cortison | 0,9% NaCl Inhalation |
| Rhinovirus* | 42 (85.7) | 40 (81.6) | 7 (14.3) | 12 (24.5) | 13 (26.5) | 31 (63.3) | 31 (63.3) | 6 (12.2) | 12 (24.5) | 2 (4.1) | 3 (6.1) | 0 (0) |
| RSV* total | 86 (96.6) | 68 (76.4) | 15 (16.9) | 43 (48.3) | 30 (33.7) | 64 (71.9) | 54 (60.7) | 24 (27.0) | 8 (9.0) | 11 (12.4) | 2 (2.2) | 4 (4.5) |
| RSV subtype A* | 8 (100) | 7 (87.5) | 1 (12.5) | 6 (75.0) | 2 (25.0) | 6 (75.0) | 6 (75.0) | 1 (12.5) | 2 (25.0) | 0 (0) | 1 (12.5) | 1 (12.5) |
| RSV subtype B* | 79 (96.3) | 61 (75.3) | 14 (17.3) | 37 (45.7) | 27 (33.3) | 58 (71.6) | 48 (59.3) | 23 (28.4) | 6 (7.4) | 11 (13.6) | 1 (1.2) | 3 (3.7) |
| Adenovirus* | 4 (36.4) | 6 (54.5) | 1 (9.1) | 10 (90.9) | 3 (27.3) | 5 (45.5) | 11 (100) | 0 (0) | 4 (36.4) | 1 (9.1) | 0 (0) | 0 (0) |
| Metapneumovirus* | 12 (85.7) | 9 (64.3) | 1 (7.1) | 9 (64.3) | 2 (14.3) | 6 (42.8) | 11 (78.6) | 2 (14.3) | 2 (14.3) | 1 (7.1) | 0 (0) | 0 (0) |
| Enterovirus* | 1 (33.3) | 2 (66.7) | 0 (0) | 3 (100) | 2 (66.7) | 2 (66.7) | 3 (100) | 0 (0) | 2 (66.7) | 0 (0) | 0 (0) | 0 (0) |
| Parainfluenza 1-3* | 10 (90.9) | 9 (81.8) | 2 (18.2) | 5 (45.5) | 6 (54.5) | 7 (63.6) | 9 (81.8) | 1 (9.1) | 1 (9.1) | 0 (0) | 0 (0) | 0 (0) |
| Influenza A, B, C* | 41 (75.9) | 31 (57.4) | 4 (7.4) | 46 (85.2) | 15 (27.8) | 24 (44.4) | 48 (88.9) | 2 (3.7) | 5 (9.3) | 2 (3.7) | 1 (1.9) | 0 (0) |
| Human Coronaviruses* | 11 (100) | 10 (90.9) | 1 (9.1) | 4 (36.4) | 5 (45.5) | 7 (63.6) | 6 (54.5) | 1 (9.1) | 1 (9.1) | 2 (18.2) | 0 (0) | 0 (0) |
| SARS COV2* | 2 (66.7) | 1 (33.3) | 2 (66.7) | 2 (66.7) | 0 (0) | 3 (100) | 2 (66.7) | 0 (0) | 0 (0) | 1 (33.3) | 0 (0) | 0 (0) |
| Negative | 45 (78.9) | 42 (73.7) | 10 (17.5) | 28 (49.1) | 14 (24.6) | 32 (56.1) | 37 (64.9) | 4 (7.0) | 5 (8.8) | 3 (5.3) | 1 (1.8) | 2 (3.5) |
| Multiple viral infections | 38 (88.4) | 34 (79.1) | 6 (14.0) | 22 (51.2) | 9 (20.9) | 33 (76.7) | 31 (72.1) | 8 (18.6) | 6 (14.0) | 2 (4.7) | 3 (7.0) | 1 (2.3) |
| Total | 292 (84.6) | 252 (73.0) | 49 (14.2) | 184 (53.3) | 99 (28.7) | 214 (62.0) | 243 (70.4) | 48 (13.9) | 46 (13.3) | 25 (7.2) | 10 (2.9) | 7 (2.0) |

Data are presented as absolute numbers and relative frequencies (%) of positive tested pathogen group

*Indicates numbers of single infections without co-infections

**Supplementary Table 2.** Modified Tal Score per pathogen category

| **Modified Tal Score** | **No bronchiolitis** | **Mild bronchiolitis** | | | | | **Moderate bronchiolitis** | | | | | **Severe bronchiolitis** | |
| --- | --- | --- | --- | --- | --- | --- | --- | --- | --- | --- | --- | --- | --- |
|  | 0 | 1 | 2 | 3 | 4 | 5 | 6 | 7 | 8 | 9 | 10 | 11 | 12 |
| Rhinovirus* | 40 (81.6) | 2 (4.1) | 0 (0) | 4 (8.2) | 3 (6.1) | 0 (0) | 0 (0) | 0 (0) | 0 (0) | 0 (0) | 0 (0) | 0 (0) | 0 (0) |
| RSV* total | 50 (56.2) | 6 (6.7) | 8 (9.0) | 6 (6.7) | 9 (10.1) | 4 (4.5) | 4 (4.5) | 1 (1.1) | 1 (1.1) | 0 (0) | 0 (0) | 0 (0) | 0 (0) |
| RSV subtype A* | 6 (75.0) | 0 (0) | 0 (0) | 1 (12.5) | 0 (0) | 0 (0) | 0 (0) | 0 (0) | 1 (12.5) | 0 (0) | 0 (0) | 0 (0) | 0 (0) |
| RSV subtype B* | 44 (54.3) | 6 (7.4) | 8 (9.9) | 5 (6.2) | 9 (11.1) | 4 (4.9) | 4 (4.9) | 1 (1.2) | 0 (0) | 0 (0) | 0 (0) | 0 (0) | 0 (0) |
| Adenovirus* | 11 (100) | 0 (0) | 0 (0) | 0 (0) | 0 (0) | 0 (0) | 0 (0) | 0 (0) | 0 (0) | 0 (0) | 0 (0) | 0 (0) | 0 (0) |
| Metapneumovirus* | 11 (78.6) | 0 (0) | 2 (14.3) | 0 (0) | 0 (0) | 0 (0) | 1 (7.1) | 0 (0) | 0 (0) | 0 (0) | 0 (0) | 0 (0) | 0 (0) |
| Enterovirus* | 3 (100) | 0 (0) | 0 (0) | 0 (0) | 0 (0) | 0 (0) | 0 (0) | 0 (0) | 0 (0) | 0 (0) | 0 (0) | 0 (0) | 0 (0) |
| Parainfluenza 1-3* | 10 (90.9) | 0 (0) | 0 (0) | 0 (0) | 0 (0) | 0 (0) | 0 (0) | 0 (0) | 0 (0) | 1 (9.1) | 0 (0) | 0 (0) | 0 (0) |
| Influenza A, B, C* | 48 (88.9) | 4 (7.4) | 0 (0) | 1 (1.9) | 0 (0) | 1 (1.9) | 0 (0) | 0 (0) | 0 (0) | 0 (0) | 0 (0) | 0 (0) | 0 (0) |
| Human Coronaviruses* | 10 (90.9) | 0 (0) | 1 (9.1) | 0 (0) | 0 (0) | 0 (0) | 0 (0) | 0 (0) | 0 (0) | 0 (0) | 0 (0) | 0 (0) | 0 (0) |
| SARS COV2* | 2 (66.7) | 0 (0) | 0 (0) | 1 (33.3) | 0 (0) | 0 (0) | 0 (0) | 0 (0) | 0 (0) | 0 (0) | 0 (0) | 0 (0) | 0 (0) |
| Negative | 52 (91.2) | 2 (3.5) | 1 (1.8) | 1 (1.8) | 1 (1.8) | 0 (0) | 0 (0) | 0 (0) | 0 (0) | 0 (0) | 0 (0) | 0 (0) | 0 (0) |
| Multiple viral infections | 27 (62.8) | 3 (7.0) | 4 (9.2) | 5 (11.6) | 2 (4.7) | 2 (4.7) | 0 (0) | 0 (0) | 0 (0) | 0 (0) | 0 (0) | 0 (0) | 0 (0) |
| Total | 264 (76.5) | 17 (4.9) | 16 (4.6) | 18 (5.2) | 15 (4.3) | 7 (2.0) | 5 (1.4) | 1 (0.3) | 1 (0.3) | 1 (0.3) | 0 (0) | 0 (0) | 0 (0) |

*Indicates numbers of single infections without co-infections

Data are presented as absolute numbers and relative frequencies (%) of positive tested pathogen group
